# Supplementary material for: An Optimized HPLC-DAD Methodology for the Determination of Anthocyanins in Grape Skins of Red Greek Winegrape Cultivars (Vitis vinifera L.)
Source: Molecules. 2022 Oct 21;27(20):7107. doi: 10.3390/molecules27207107 (PMC9610061; doi:10.3390/molecules27207107)
Supplement: Supplementary file 1 [file molecules-27-07107-s001.zip › molecules-1957888-supplementary.pdf]

Supplementary Material

An Optimized HPLC-DAD Methodology for the Determination of Anthocyanins in Grape Skins of Red Greek winegrape Cultivars (*Vitis vinifera* L.)

Natasa P. Kalogiouri, Christina Karadimou, Mary S. Avgidou, Elissavet Petsa, Emmanouil-Nikolaos Papadakis, Serafeim Theocharis, Ioannis Mourtzinos, Urania Menkissoglu-Spiroudi and Stefanos Koundouras

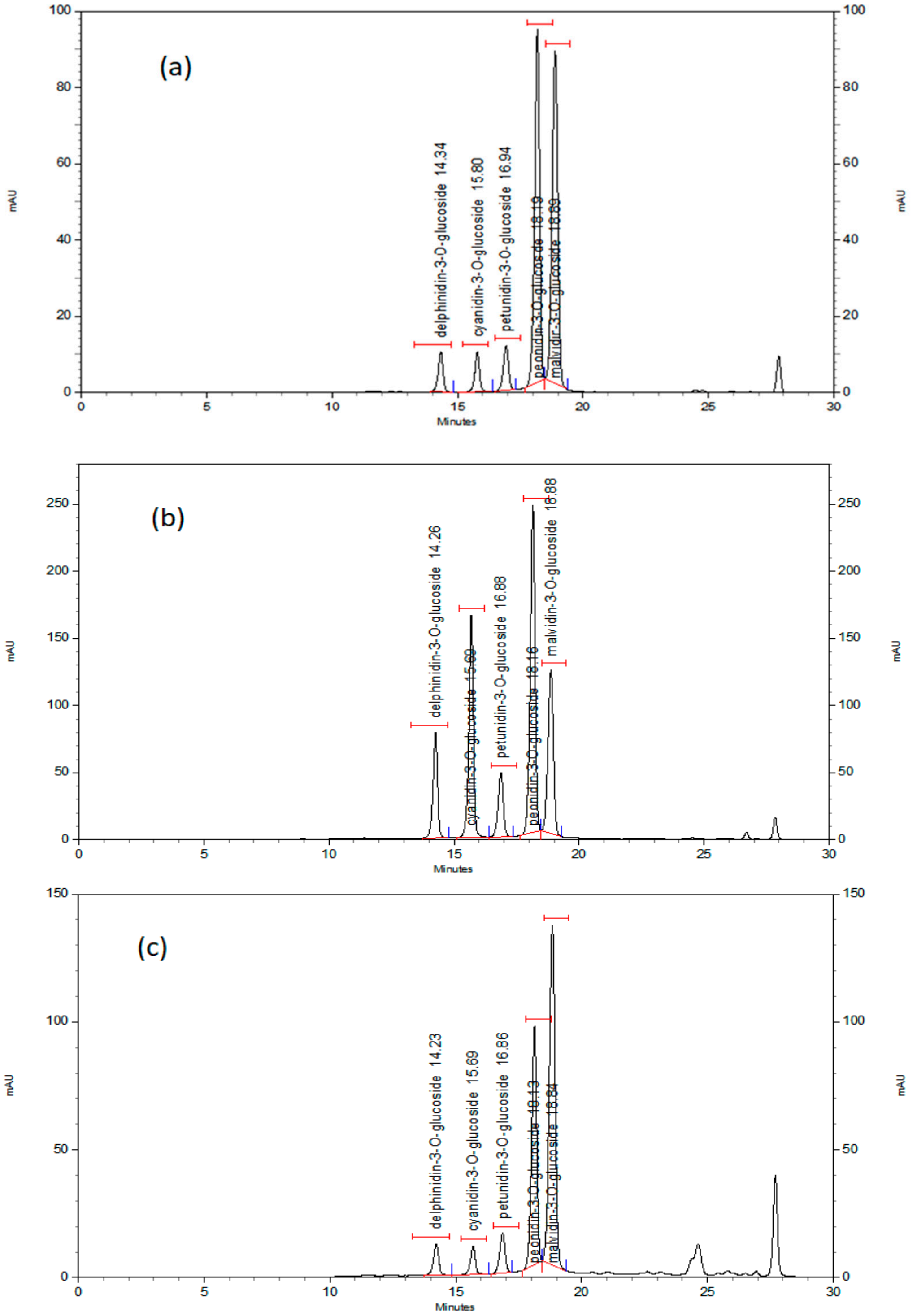

Figure S1. Characteristic chromatograms of real non-spiked samples, one of each variety: (a) Vradiano; (b) Kotsifali; (c) Limnio.

Table S1. Percentage of individual anthocyanin content in the skins of the red grape varieties 'Kotsifali', 'Limnio' and 'Vradiano'.

	**Dlp**	**Cyn**	**Pt**	**Pn**	**Mlv**
**A1**	6.9	8.3	6.2	35.7	43.0
**A2**	11.1	15.4	7.7	35.3	30.4
**A3**	10.9	20.4	7.2	35.7	25.8
**A4**	9.1	17.5	6.8	34.4	32.2
**A5**	6.7	15.7	6.6	32.4	38.7
**A6**	7.9	7.7	7.0	36.5	40.9
**B1**	3.6	1.0	4.6	13.6	77.3
**B2**	2.6	1.4	3.2	24.3	68.5
**B3**	3.4	1.8	4.0	27.9	63.0
**B4**	3.7	1.8	4.2	22.2	68.1
**B5**	2.6	1.1	3.3	15.6	77.4
**B6**	4.0	3.7	4.6	29.7	58.0
**B7**	2.9	1.6	3.5	14.0	78.1
**C1**	4.5	3.8	4.6	37.3	49.9
**C2**	3.7	5.2	4.1	48.8	38.1
**C3**	5.6	4.3	5.8	39.5	44.9

Dlp: delphinidin-3-O-glucoside; Cyn: cyanidin-3-O-glucoside; Pt: petunidin-3-O-glucoside; Pn: peonidin-3-O-glucoside; Mlv: malvidin-3-O-glucoside

Table S2. Classical analysis results of red grapes

Sample	Variety	Region	Brix	pH	Total Acidity (g/L tartaric acid)	weight of 50 grapes (g)
A1	Kotsifali	Heraklion	21.8	3.5	5.9	107.5
A2	Kotsifali	Heraklion	23.9	3.4	6.8	98.6
A3	Kotsifali	Heraklion	18.5	3.1	7.8	110.2
A4	Kotsifali	Heraklion	23.1	3.2	6.5	98.5
A5	Kotsifali	Athens	20.4	3.5	6.8	76.4
A6	Kotsifali	Thessaloniki	26.0	3.7	6.5	102.6
B1	Limnio	Athens	19.3	3.7	3.9	51.6
B2	Limnio	Thessaloniki	22.4	3.4	5.6	135.0
B3	Limnio	Halkidiki	22.3	3.7	4.4	91.4
B4	Limnio	Halkidiki	21.0	3.4	5.3	76.8
B5	Limnio	Xanthi	21.5	3.4	5.6	93.7
B6	Limnio	Lemnos	21.7	3.8	4.2	58.8
B7	Limnio	Serres	21.2	3.5	4.8	106.0
C1	Vradiano	Athens	19.7	3.5	6.0	82.5
C2	Vradiano	Euboea	22.9	3.4	4.8	123.8
C3	Vradiano	Euboea	23.0	3.4	4.8	120.8
